# A short peptide of the C-terminal class Y helices of apolipoprotein A-I has preserved functions in cholesterol efflux and in vivo metabolic control

**DOI:** 10.1038/s41598-020-75232-0

**Published:** 2020-10-22

**Authors:** Shelley J. Edmunds, Rebeca Liébana-García, Karin G. Stenkula, Jens O. Lagerstedt

**Affiliations:** 1 Biomedical Center Floor C13, Lund University Diabetes Center, Tornavagen 10, 221 84 Lund, Sweden; 2 Lund Institute of Advanced Neutron and X-ray Science (LINXS), Lund, Sweden

**Keywords:** Biochemistry, Endocrinology, Endocrine system and metabolic diseases, Molecular medicine

## Abstract

Apolipoprotein A-I (ApoA-I) of high-density lipoprotein (HDL) induces glucose uptake by muscle tissues and stimulates pancreatic insulin secretion, and also facilitates cholesterol transport in circulation, and is explored for anti-diabetic and anti-atherosclerotic treatments. As the better alternative to complex protein–lipid formulations it was recently established that the C-terminal region of the ApoA-I protein singly improves the metabolic control and prevents formation of atherosclerotic plaques. Additional investigations of peptides based on the ApoA-I structure may lead to novel anti-diabetic drugs. We here investigate a short peptide (33mer, RG33) that corresponds to the two last helical segments (aa 209–241) of the ApoA-I structure (so-called class Y-helices which forms amphipathic helices) for stability and solubility in serum, for in vitro cholesterol efflux capability, and for providing in vivo glucose control in an insulin resistant mouse model. The RG33 peptide efficiently solubilizes lipid-vesicles, and promotes the efflux of cholesterol from cultured macrophages. The efflux capacity is significantly increased in the presence of lipids compared to non-lipidated RG33. Finally, acute treatment with the RG33 peptide significantly improves the glucose clearance capacity of insulin resistant mice. The impact of the RG33 peptide on glucose control and cholesterol transport, as well as the physicochemical properties, makes it a good candidate for translational exploration of its therapeutic potential in diabetes treatment.

## Introduction

Type 2 diabetes (T2D) remains one of the major diseases worldwide^1^ and involves insufficient amounts of secreted postprandial insulin as well as insulin resistance at peripheral tissues such as muscle, liver and fat^2^. Diabetes is also strongly linked to cardiovascular diseases^3,4^. Understanding the biology that connects the two diseases, as well as finding new therapeutics, is of great importance for the affected individuals.

High-density lipoprotein (HDL) mediates the uptake and transport of cholesterol and lipids from the periphery to the liver, a process referred to as the reverse cholesterol transport pathway. In the liver, the cholesterol is either reused, or catabolized and excreted as bile^5^. Excess accumulation of cholesterol at various sites has a negative impact on the cardiovascular health of individuals and it is directly connected to plaque formation in atherosclerosis^6^. As the main protein component of HDL, apolipoprotein A-I (ApoA-I) has a central role in the reverse cholesterol pathway.

ApoA-I also affects glucose homeostasis^7–12^, which occurs via increased insulin secretion by beta cells of the pancreas^7,13–15^ and also by direct stimulation of skeletal and heart muscles to increase their glucose uptake^10,11,14,16^. Collectively, the findings suggest that ApoA-I and HDL have novel and specific roles in the regulation of glucose control. This notion is further supported by a recent clinical study that concludes that individuals with low HDL levels have an increased risk to develop T2D due to a decline in beta cell function over time^17^.

Investigations on the specific functions of ApoA-I in glycemic control have identified the C-terminal domain of the ApoA-I protein (amino acid residues 190–243) as the bioactive domain in glucose control^11^. Based on this, the RG54 peptide was verified for enhanced in vivo glucose control (prediabetic and diabetic DIO and db/db mice) and prevention of atherosclerosis (apoE-/- mice), as well as for improved in vitro beta cell insulin secretion, macrophage cholesterol efflux and muscle glucose uptake (rodent and human myotubes)^15^. While these are important findings, the size of the RG54 peptide makes it less attractive for further exploration as T2D therapeutics. A short peptide (33mer, RG33) that corresponds to the two last helical segments (aa 209–241) of the ApoA-I structure is here analyzed for stability and solubility in serum, for in vitro cholesterol efflux capability, and for in vivo glucose control in an insulin resistant mouse model.

## Materials and methods

### Synthesis, formulation and quality control of synthetic peptides

Peptide (lyophilized powder) produced by Red Glead AB (Lund, Sweden) was reconstituted as previously described^15^. Peptide concentration was determined using a NanoDrop 2000c spectrophotometer (Thermo Scientific), and integrity was analysed by Tris-Tricine SDS-PAGE followed by Coomassie staining and by circular dichroism spectroscopy (CD). CD spectra were acquired on a Jasco J-810 spectropolarimeter equipped with a Jasco CDF-426S Peltier, set to 25 °C^18^. Samples were loaded into a 1 mm quartz cuvette and CD spectra acquired at 25 °C in the far-UV range 200–260 nm, with a 1 nm wavelength increment. Averages of five scans were baseline-subtracted (PBS buffer; 25 mM phosphate, 150 mM NaCl) and the alpha-helical content was calculated from the molar ellipticity at 222 nm as previously described^11,19^. Human plasma (citrate) was from Blodcentralen at the Uppsala academic hospital and pooled from four healthy volunteer blood donors after giving informed consent for general research use. All methods were carried out in accordance with relevant guidelines and regulations, and all experimental protocols were approved by the Science for Life Laboratories at Uppsala University, Sweden.

Mouse CD-1 plasma (heparin) was from Novakemi AB. Analysis was performed using LC–MS/MS, a Sciex QTRAP 6500 coupled to a Waters Acquity LC system with an attached HSS T3 2 × 50 mm column. A 5%-100% acetonitrile gradient and 0.1% formic acid was used as mobile phase.

### In silico peptide analyses

The online software “*NetWheels: Peptides Helical Wheel and Net projections maker*” available at https://lbqp.unb.br/NetWheels/ was utilized for the generation of the helical wheel depiction^20^.

### In vitro cholesterol efflux assay

The mouse macrophage-like cell line, J774 (ATCC TIB-67), loaded with ^3^H-cholesterol, was used for cholesterol efflux to acceptors (RG33 peptide, lipid-free or lipid-bound) as previously described^21^. For this, J774 macrophages (ATTC) were plated into cell culture dishes at 80,000 cells/well (500 µl/well) in RPMI 1640 (Thermo scientific) supplemented with 10% FBS and gentamicin. The media was after 24 h replaced with 250 µl/well RPMI 1640 containing 5% FBS, 4 µCi/ml ^3^H-cholesterol (Perkin Elmer), 2 µg/ml ACAT inhibitor (Sandoz 58–035, Sigma) and gentamicin. After an additional 24 h the ^3^H-cholesterol media was replaced with 500 µl RPMI 1640 supplemented with 0.2% BSA (low free fatty acids and low endotoxin, Sigma), 2 µg/ml ACAT inhibitor, 0.3 mmol/l Cpt-cAMP (Abcam) and gentamicin for 24 h. The cells were washed twice with RPMI 1640 containing gentamicin and then duplicate, or triplicate, wells were treated with 300 µl/well cholesterol acceptor (LF RG33, or LB RG33 at a molar protein-to-lipid ratio of 1:100) in RPMI 1640 containing gentamicin at the indicated concentrations. Collected media (200 µl) was centrifuged at 14,000×*g* for 5 min at room temperature to pellet any collected cells, and 100 µl supernatant transferred to a scintillation vial. 5 ml of scintillation fluid was added to each sample before scintillation counting was performed on a Wallac Guardian 1414 Liquid Scintillation Counter (Perkin Elmer). To obtain a measure of ^3^H-cholesterol in the cells before efflux incubations began a set of wells from each experiment were incubated with 1% sodium deoxycholate and the lysate collected for scintillation counting. The mean reading from these samples was considered to be representative of the total cellular pool of ^3^H-cholesterol available for efflux to RG33 and therefore total efflux for each treatment was calculated as a % of this value. Non-specific background efflux was measured in triplicate for all relevant time points in each experiment and subtracted from time-matched treatments. The ACAT inhibitor and CPT-cAMP were used to prevent formation of cholesterol esters of the ^3^H-cholesterol and to induce expression of ABCA1, respectively.

### Recombinant HDL formation

1,2-Dimyristoyl-sn-glycero-3-phosphocholine (DMPC; Avanti Polar Lipids) was formed into 100 nm multilamellar vesicles (MLV) via extrusion using the LiposoFast system (Avestin) as previously described^11^. MLV were incubated with RG33 at indicated ratios for 4 days at 24 °C to form rHDL. The resulting particle sizes were measured by Dynamic Light Scattering using a Zetasizer APS (Malvern Instruments).

### In vivo—glucose control

Male C57BL/6NTac mice were purchased from Taconic (Ejby, Denmark) at 8–9 weeks of age, acclimatised for one week on normal chow diet, then changed to a High-Fat Diet (HFD; Research Diets D12492, 60% fat content) for 2 weeks to induce insulin resistance as previously described^15^, followed by glucose tolerance testing (GTT) as indicated (Fig. 4a). For the GTTs, mice (n = 6) fasted overnight (9 h) were injected with the RG33 peptide or saline control (data for saline controls are adapted from^15^), and after an additional 3 h given intraperitoneal (i.p.) glucose (40 mg/mouse) followed by collection of serum samples at the indicated times. Blood glucose levels were measured immediately (OnetouchUltra2, Lifescan) and insulin levels were assayed in serum using ELISA (Mercodia). Male mice only were chosen for these studies as they have been shown to develop more severe and reproducible clinical symptoms of diabetes than females in these models^22^. All animals were housed in conventional shoebox cages with standard wood chip bedding, 3–5 animals/cage, maintained in a humidity-controlled room with a 12-h light/dark cycle, and had non-restricted food and water. Cages were changed twice weekly and food and water refreshed every two days. All peptides were dissolved in sterile 0.9% saline to give injection volumes of 0.1–0.2 ml/mouse, and injected i.p. or subcutaneous (subQ) as these are the expected injection routes for human usage. Mice were euthanised via cervical dislocation. All animal procedures were approved by the Malmö/Lund Committee for Animal Experiment Ethics, Lund, Sweden, and all experiments were performed in accordance with their relevant guidelines and regulations.

### Statistics

All numerical data are presented as mean ± SD or SEM. Significance was calculated by unpaired, two-tailed Student’s T-test, or one-way ANOVA with Dunnett’s post hoc multiple comparison using GraphPad Prism (version 8.0). *p* < 0.05 was considered significant.

## Results and discussion

**Figure 1:**
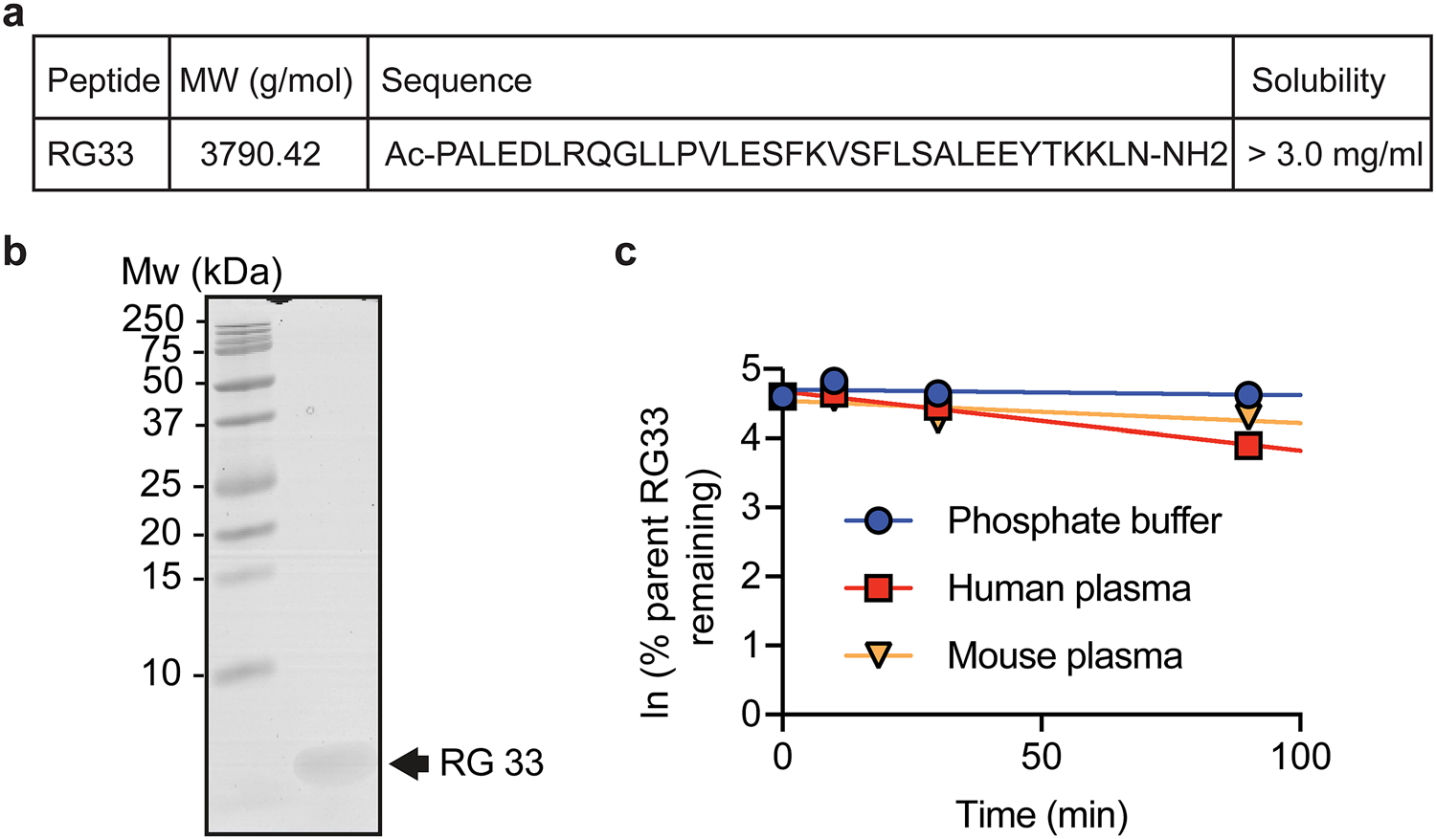
Peptide characterization for RG33. (**a**) Full peptide sequence. (**b**) Peptide purity as shown by Tris/Tricine SDS PAGE visualized by coomassie staining, 5 μg/well. (**c**) Stability of native or refolded RG33 over time in phosphate buffer, human plasma or mouse plasma. GraphPad Prism (version 8.0) (www.graphpad.com) was used.

A 33-mer peptide (RG33) that corresponds to the two Class Y helices (11 and 22 residues, respectively) of the C-terminal domain of ApoA-I (corresponding to regions 209–219 and 220–241 of ApoA-I, respectively) was synthesized and chemically modified at the termini for increased stability (Fig. 1a), and analyzed by SDS-PAGE (Fig. 1b; Suppl. Fig.). Such modifications are known to lead to loss of the terminal charges of the modified peptide with potential reduction in solubility. However, the solubility of the RG33 peptide was found to be sufficiently high (> 3.0 mg/ml). Moreover, the stability of the peptide was tested by incubation for up to 90 min in either PBS or human or mouse plasma followed by LC–MS analysis of the peptide in the complex mixture, or for longer time periods (up to 168 h at 5 °C or 22 °C) in PBS followed by LC-UV analysis of the pure peptide. When solubilized in PBS, the RG33 peptide was intact in both the shorter incubation scheme and during longer storage at both 5 °C and 22 °C for up to a week (not shown), whereas incubation with plasma (human and mouse) led to some degradation of the RG33 peptide within the 90 min time frame (Fig. 1c). The extent of degradation in plasma was assumed not to affect the in vitro and in vivo analyses made herein. Nevertheless, while not needed for the current study, further modifications aimed at prolonging the half-life of the RG33 peptide in plasma would be beneficial for therapeutic uses.

**Figure 2:**
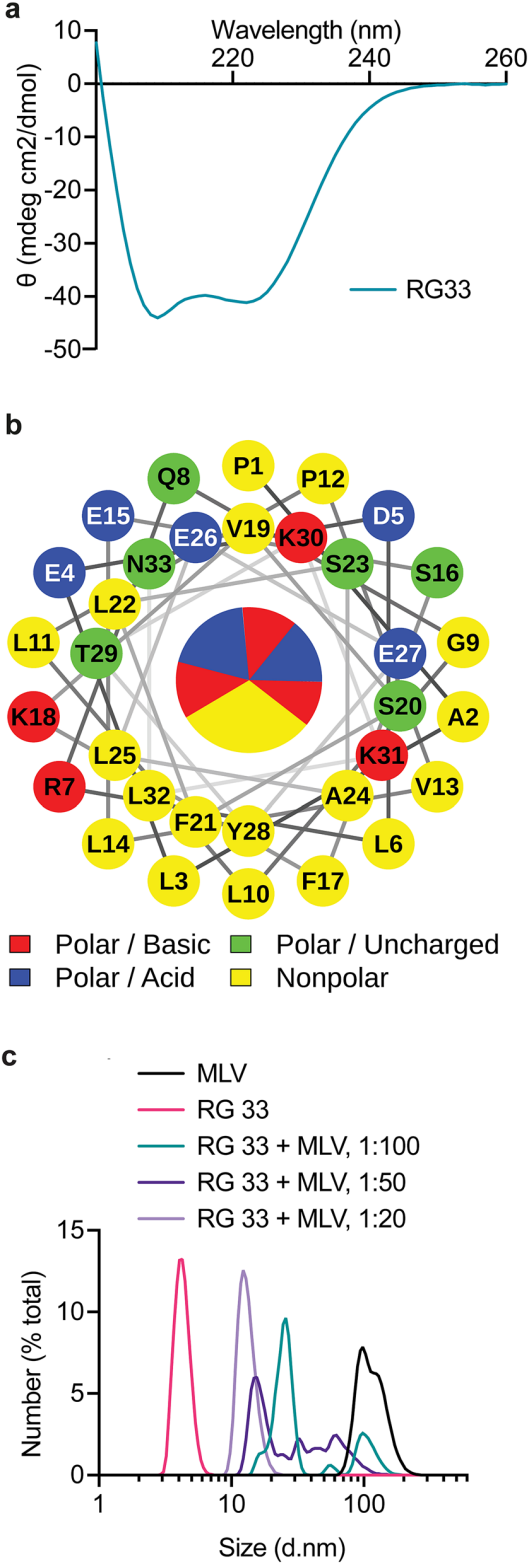
Peptide structure and lipid complex formation activity of RG33. (**a**) Circular dichroism spectroscopy of RG33 dissolved in PBS at 0.2 mg/ml. This spectrum represents the average of five measurements using five separate solutions. (**b**) Helical wheel analysis of residues 209 to 241 of the ApoA-I protein. One-letter code for amino acids is used and numbered 1 to 33 with start at residue 209. (**c**) rHDL particles were prepared by incubating the MLVs with RG33 peptide in the molar ratios noted at 24 °C for four days. Particle size distribution was analysed by DLS and frequency curves generated for RG33. Both MLVs alone (data on pure 100 nm MLVs is adapted from^15^) and lipid-free peptide are included as controls. GraphPad Prism (version 8.0) (www.graphpad.com) was used. Data represents five individual experiments.

Circular dichroism (CD) spectroscopy (Fig. 2a) was next used to assay for peptide integrity and secondary structure. The helical content of the RG33 peptide in solution was based on the CD data and estimated to be on average 31%. This is higher than the helical content of the 190–243 ApoA-I peptide which was previously shown to be in the range of 17–21% alpha-helix^11^. A high helical content combined with an amphipathic arrangement of the helices is important for the lipid-binding function. Indeed, the binding of lipids induces formation of amphipathic helixes of the ApoA-I protein^23^. In line with this, helical wheel analysis of the RG33 peptide (Fig. 2b) showed a sidedness of the physicochemical properties of the amino acid side chains with one side being nonpolar, this side is expected to be in peptide-lipid or peptide-peptide interactions, and one side being polar with shifting basic-acidic character. As originally described by Segrest and colleagues^24^ and recently discussed by Oda^25^, this alternating arrangement of polar (basic/acidic/uncharged) and nonpolar residues is typical for Class Y helices.

As described above, the amphipathic organization of the RG33 peptide is expected to promote efficient lipid-binding. To asses this function we incubated the RG33 peptide with multilamellar vesicles (MLVs), consisting of DMPC phospholipids, at different peptide-to-lipid ratios followed by analysis of the hydrodynamic radii by dynamic light scattering (DLS) technology. The apparent size distributions of the formed peptide-lipid complexes were compared with those of pure RG33 peptides (this study) and of pure 100 nm MLVs (data adapted from^15^). As can be seen (Fig. 2c), complete solubilization and encapsulation of the DMPC lipids occur only at the 1:20 peptide-to-lipid molar ratio, whereas the 1:50 molar ratio show only partial solubilization. The amount of RG33 peptide needed to fully solubilize MLVs is thus higher than that of both the RG54 peptide (region 190–243 of the ApoA-I structure) and the full-length ApoA-I (residues 1–243), which form homogenous 10–20 nm complexes already at peptide-to-lipid molar ratios of 1:50 and 1:100, respectively, as previously described^15^. However, given the size differences, with 7–8 units of the RG33 peptides corresponding to one full-length ApoA-I protein, it may be argued that the observed differences in solubilization efficiencies are not as extensive as they appear but rather reflects that a larger number of molecules of the shorter peptides are need to encapsulate the same number of lipids. Independent of which, the findings encouraged us to evaluate the catalytic efficiencies of the RG33 peptide in the lipid-free (LF) and lipid-bound (LB) states.

**Figure 3:**
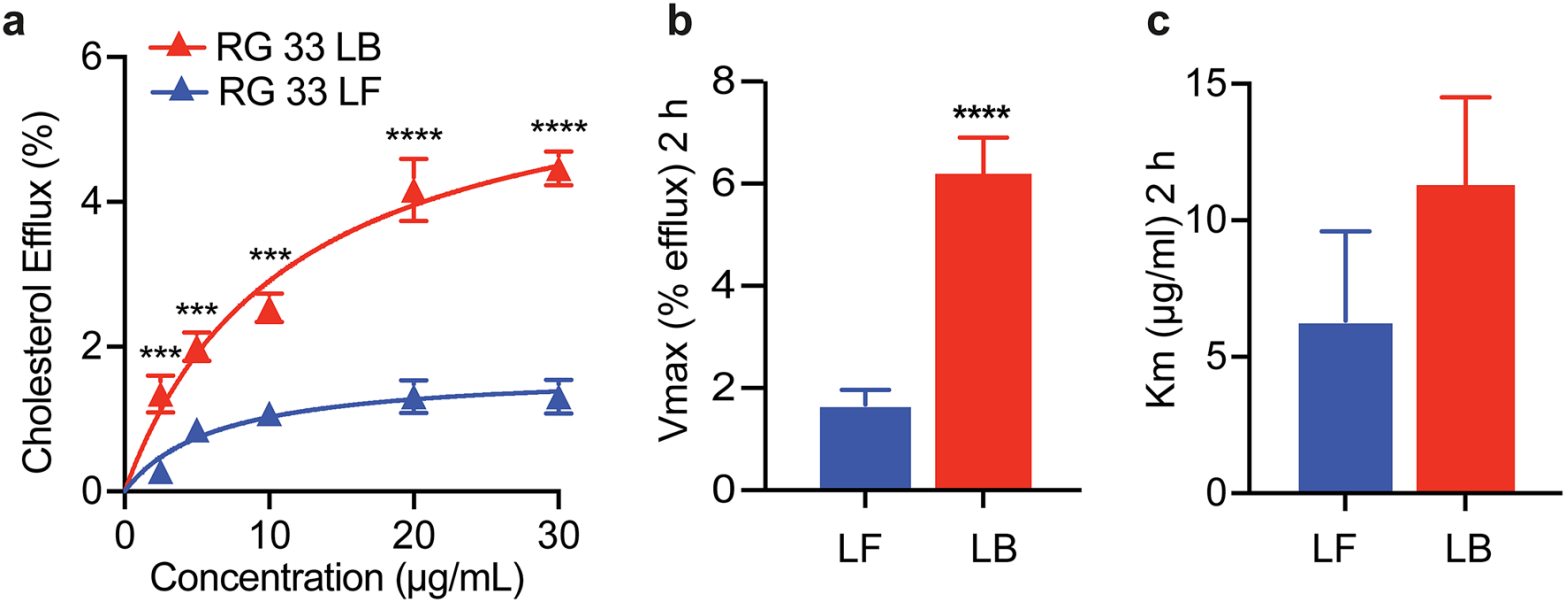
Cholesterol efflux activity of RG33 in lipid-free (LF) and lipid-bound (LB) states. (**a**) Concentration dependence of cholesterol efflux from ^3^H-cholesterol-labeled J774 cells by lipid-free (LF) and lipid-bound (LB) RG33 was compared after 2 h, n = 3. The maximum rate (V_max_) (**b**) and the Michaelis constant (K_m_) (**c**) were calculated using the Michaelis–Menten equation. GraphPad Prism (version 8.0) (www.graphpad.com) was used. Values are means ± SEM, ***p ≤ 0.001, ****p ≤ 0.0001.

To analyze this, cultured J774 macrophages were loaded with isotope-labelled cholesterol, followed by incubation with RG33 (LF or LB) at specified concentrations for 2 h (Fig. 3a). Both LF and LB RG33 catalyzed a concentration dependent efflux of cholesterol, with the level of effluxed cholesterol catalyzed by LF RG33 being comparable to the that previously described for LF RG54 peptide^15^ and significantly higher for LB RG33 compared to LF RG33 at all concentrations. The markedly increased capacity was also reflected in a threefold higher Vmax for the LB RG33 compared to the LF RG33 (6.2 vs 1.7% efflux at 2 h, respectively; *p* ≤ 0.0001) (Fig. 3b), which is a larger difference than previously observed for the RG54 peptide (twofold increase for LB vs LF RG54 as previously described^15^). No significant difference in binding efficiency (Km) between LF and LB RG33 was observed (Fig. 3c). In summary, and of potential significance for therapeutic potential for cardiovascular diseases, these data show that the lipid-binding and cholesterol efflux capacity previously described for the RG54 peptide^15^ is preserved in the shorter RG33 peptide.

**Figure 4:**
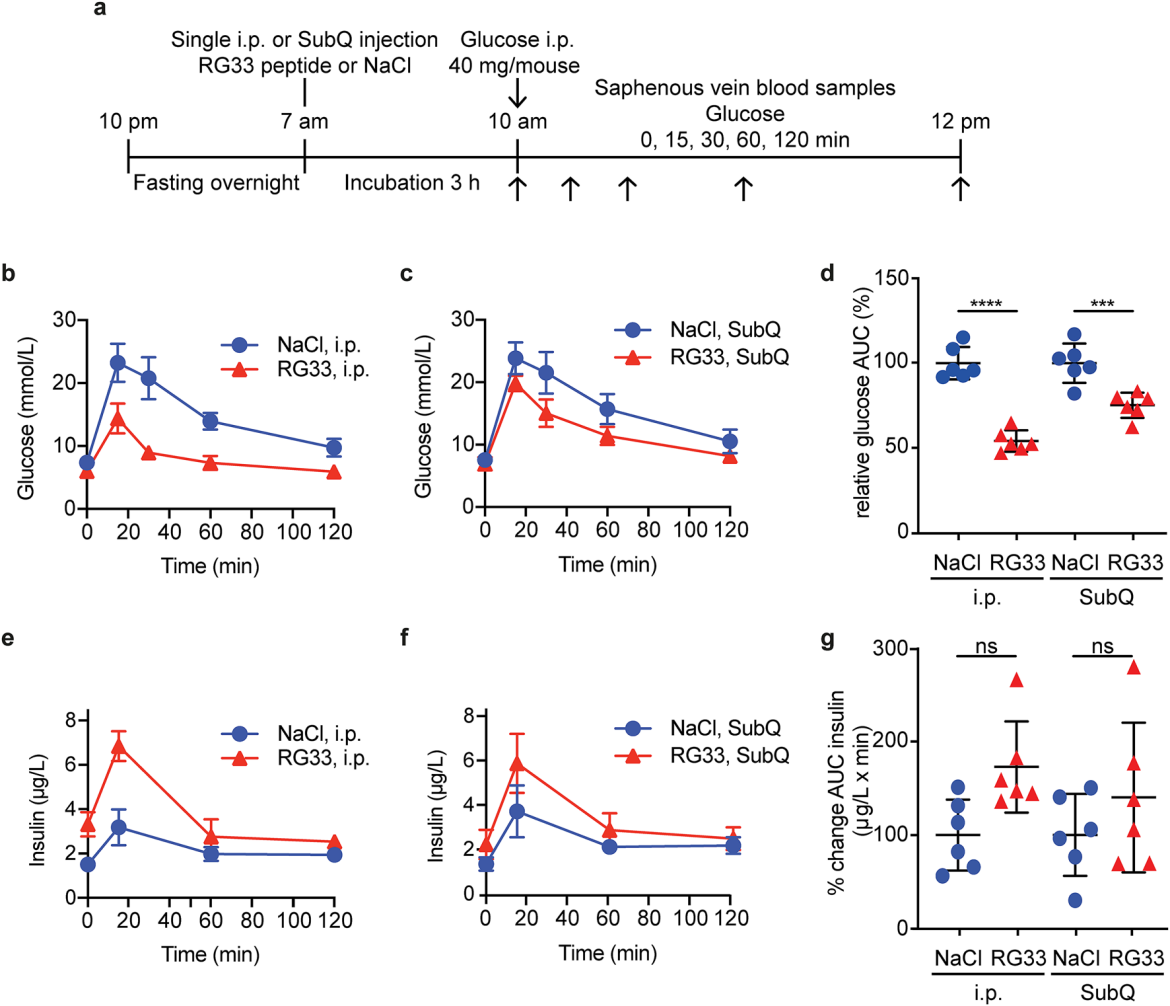
RG33 improves glucose tolerance in GTT of DIO mice when administered by intraperitoneal or subcutaneous injection. (**a**) Male C57BL/6 mice fed High-Fat Diet for two weeks were fasted overnight, then treated for 3 h with a single intraperitoneal injection (i.p.), or subcutaneous injection (subQ) of NaCl (negative control, 200 µl, n = 6), or RG33 (12 mg/kg, n = 6). Mice then received an i.p. glucose load (40 mg/mouse) followed by blood and plasma collection at the indicated times and blood glucose (**b**, **c**) and insulin (**e**, **f**) concentrations determined for both injection routes. Total AUC glucose and AUC insulin were calculated (mmol/l x min and μg/l x min, respectively) then normalised to the average values of the relevant NaCl control groups to give relative AUC (% control) for glucose (**d**) and insulin (**g**). Data for NaCl control groups are adapted from^15^. GraphPad Prism (version 8.0) (www.graphpad.com) was used. Values are means ± SD, ***p ≤ 0.001, ****p ≤ 0.0001 versus NaCl control.

We next wished to analyze if the preserved functions of the RG33 peptide also extended to glucose control. Earlier analyses of the larger RG54 peptide included pre-diabetic (diet-induced obese (DIO) mice) and diabetic db/db rodent models, as well as cultured pancreatic beta cells and muscle myotubes^15^, which allowed for detailed analysis of tissue specific effects. Since we also wished to assay for the overall efficacy of the shortened RG33 peptide in providing systemic in vivo glucose control (and compare that to RG54) we used DIO mice with established insulin resistance^15^. The studies were also designed so that we could compare experimental i.p. injection of the RG33 peptide with SubQ administration. As described in Fig. 4a, male C57BL/6 mice that had been on high-fat diet for two weeks where treated with a single dose of RG33 peptide (i.p. or SubQ) after an overnight fast. Three hours later, glucose tolerance tests were performed and blood glucose values were determined for the RG33 treated animals against NaCl controls (data for NaCl also previously published^15^). Both i.p. (Fig. 4b) and SubQ (Fig. 4c) administration routes of the RG33 peptide significantly reduced the plasma glucose levels (Fig. 4d). The extent of the reduction in blood glucose level was largest for the i.p. administered animals (46% lower AUC for the RG33 treated group vs controls) but still substantial for the animal group that received the RG33 peptide by SubQ administration (25% lower AUC for the RG33 treated group vs controls). The difference in blood glucose level reductions between i.p. and SubQ administration of RG33 is similar to that observed for the RG54 peptide^15^. Pharmacokinetic analyses would be needed to explain in detail but it is plausible that it is due to differences in rate of biodistribution with a slower release of RG33 peptide from the subcutaneous depots. A trend of increased insulin secretion was observed in particular in the i.p. treatment group but this was not statistically significant (Fig. 4e–g).

In summary, exploration of ApoA-I and HDL as therapeutics in cardiovascular disease and for metabolic control has, despite many attempts, not yet resulted in any viable treatment (reviewed in^26,27^). The RG33 peptide, which corresponds to the C-terminal residues 209–241 of the ApoA-I protein, efficiently promotes cholesterol efflux from macrophages and provide systemic glucose control in an insulin resistant mouse model. The functionality as cholesterol acceptor is largely improved in the presence of phospholipids, which likely involves increase formation of amphipathic helices. Formulations of the RG33 peptide with lipids may thus be beneficial for in vivo functionality, and may have the added effect of increased presence in circulation as was recently described for a short ApoA-I mimetic peptide^28^. The size of the RG33 peptide is comparable to insulin and GLP1 peptides used in the clinical setting, however further optimization of the RG33 peptide for therapeutic purposes may include size reduction as well as selected amino acid substitutions. The RG33 peptide might prove to be a viable therapeutic strategy in diabetes and cardiovascular disease, however extensive analyses of the specific mechanisms of action, as well as in depth preclinical testing, will still be required.

## Supplementary information

Supplementary Information.

## Abbreviations

CVD: Cardiovascular diseases
CD: Circular dichroism
DIO: Diet induced obesity
DLS: Dynamic light scattering
GTT: Glucose tolerance test
i.p.: Intraperitoneally
MLV: Multilamellar vesicles
RG33: Peptide corresponding to amino acid residues 209–241 of ApoA-I protein
RG54: Peptide corresponding to amino acid residues 190–243 of ApoA-I protein
SubQ: Subcutaneous
