# Supplementary material for: A short peptide of the C-terminal class Y helices of apolipoprotein A-I has preserved functions in cholesterol efflux and in vivo metabolic control
Source: Sci Rep. 2020 Oct 22;10:18070. doi: 10.1038/s41598-020-75232-0 (PMC7582918; doi:10.1038/s41598-020-75232-0)

Shelley J. Edmunds, Rebeca Liébana-García, Karin G. Stenkula, and Jens O. Lagerstedt: ***A short peptide of the C-terminal class Y helices of apolipoprotein A-I has preserved functions in cholesterol efflux and in vivo metabolic control.***

Original SDS-PAGE gel

The section included in Figure 1 of the manuscript is boxed in red.

Green box indicates the section of the same gel that was prevoiusly published in Edmunds *et al* Diabetologia 2019 (doi: 10.1007/s00125-019-4877-2) as Electronic Supplementary Material (ESM) Figure 3b.

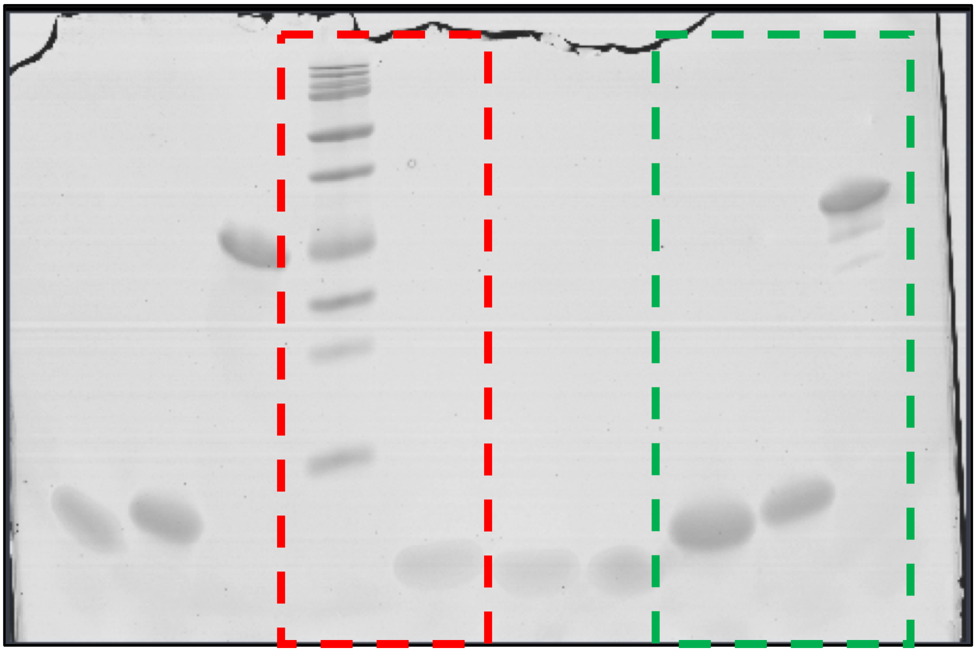

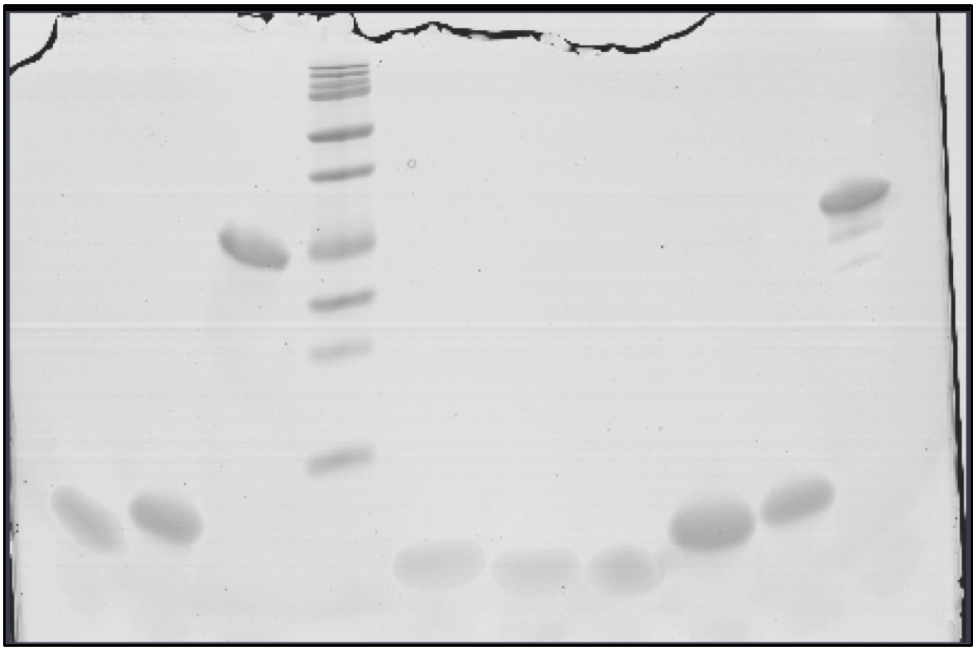

Supplement: Supplementary file 1 — Supplementary Information. [file 41598_2020_75232_MOESM1_ESM.pdf]
